# Supplementary material for: Giant magnetoelectric effects achieved by tuning spin cone symmetry in Y-type hexaferrites
Source: Nat Commun. 2017 Sep 12;8:519. doi: 10.1038/s41467-017-00637-x (PMC5595903; doi:10.1038/s41467-017-00637-x)
Supplement: Supplementary file 1 — Supplementary Information [file 41467_2017_637_MOESM1_ESM.pdf]

Type of file: pdf

Title of file for HTML: Supplementary Information

Description: Supplementary Figures, Supplementary Tables

Type of file: pdf

Title of file for HTML: Peer Review File

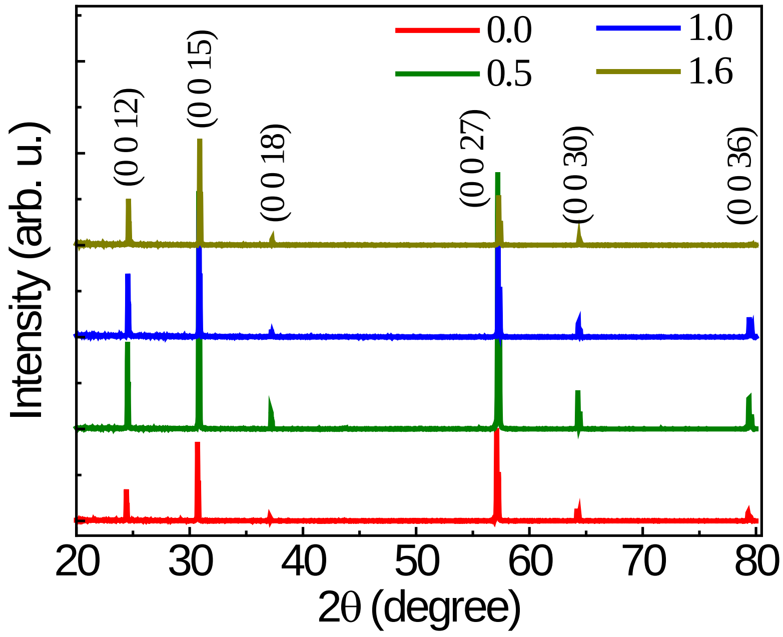

Supplementary Figure 1. XRD patterns of $Ba_{2-x}Sr_xMg_2Fe_{12}O_{22}$ samples. The XRD patterns of single crystals of $Ba_{2-x}Sr_xMg_2Fe_{12}O_{22}$ with nominal x = 0.0, 0.5, 1.0, and 1.6 at room temperature. No impurity phase is detected.

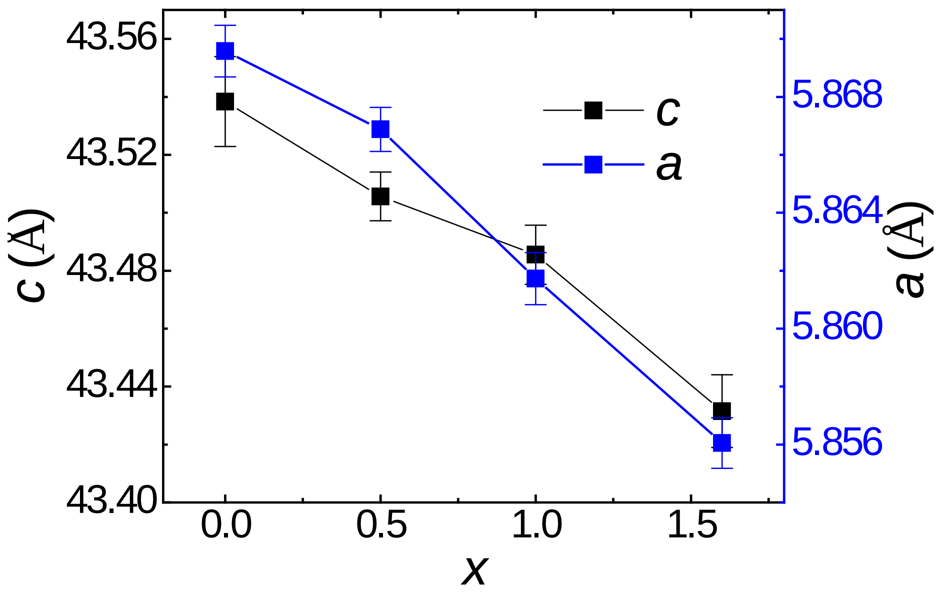

Supplementary Figure 2. Variation of the lattice constants *a* and *c* of $Ba_{2-x}Sr_xMg_2Fe_{12}O_{22}$. The change of lattice constant with nominal Sr content (x = 0.0, 0.5, 1.0, and 1.6) obtained from single crystal x-ray diffraction data. The error bar is the least-squares fit of over thousands of reflections data with a signal-to-noise-ratio of 20.

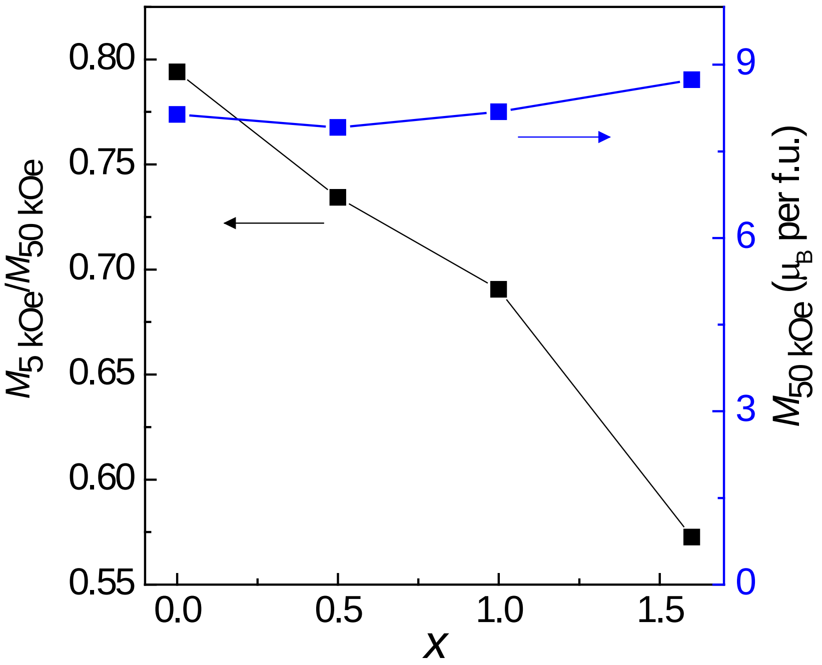

Supplementary Figure 3. The variation of magnetization with nominal Sr content *x* in $Ba_{2-x}Sr_xMg_2Fe_{12}O_{22}$. The black square represents the value of $M_{5\ kOe}/M_{50\ kOe}$ and the blue squares denote the saturation magnetization at 50 kOe.

Layer	Ion	*x*	*y*	*z*	B (Å^2)	Occ.	Mult.
1	Fe	0	0	0	0.3	1/12	1
2	O	0.1586(8)	-0.1586(8)	0.0289(1)	0.5	1/2	6
3	Ba	0	0	0.2978(2)	0.3	0.042	2
3	Sr	0	0	0.2978(2)	0.3	0.122	2
4	Fe	0	0	0.3761(1)	0.3	1/6	2
5	Fe	0	0	0.0650(1)	0.3	1/6	2
6	O	0	0	0.4192(2)	0.5	1/6	2
7	O	0.8298(8)	-0.8298(8)	0.0844(1)	0.5	1/2	6
8	Fe	0.5033(6)	-0.5033(6)	0.1094(1)	0.3	0.357	6
8	Mg	0.5033(6)	-0.5033(6)	0.1094(1)	0.3	0.143	6
9	O	0	0	0.1967(2)	0.5	1/6	2
10	O	0.1756(8)	-0.1756(8)	0.1381(1)	0.5	1/2	6
11	Fe	0	0	0.1525(1)	0.3	1/6	2
12	Fe	0	0	1/2	0.3	0.059	1
12	Mg	0	0	1/2	0.3	0.024	1

Supplementary Table 1. The crystal structure of $Ba_{0.4}Sr_{1.6}Mg_2Fe_{12}O_{22}$ refined with neutron scattering at T = 4 K. Space group $R\bar{3}m$: $a = b$ = 5.841(3) Å, c = 43.256(8) Å. χ^2 = 0.817, R_{factor} = 0.053. The initial structural model was taken from the X-ray room temperature structure with occupancy fixed. Occupancy is the multiplicity of the special position divided by the multiplicity of the general position, in this case 36.

Atomic Site	Multi.	Occupancy				
		x=0.0	x=0.5	x=1.0	x=1.5	x=1.6
Ba(3)	2	2(0)	1.72(2)	1.05(2)	0.89(2)	0.54 (3)
Sr(3)	2	0(0)	0.28(2)	0.95(2)	1.11(2)	1.46(3)
Mg/Fe(12)	1	0.29(2)	0.41(1)	0.23(2)	0.30(2)	0.29(1)
Mg/Fe(8)	6	1.71(2)	1.60(1)	1.77(2)	1.70(2)	1.71(1)
χ^2		4.54	2.07	1.92	2.32	0.85
R_{factor}		6.79	6.92	6.28	6.06	5.62

Supplementary Table 2. Occupation on Sr/Ba site change with nominal x in $Ba_{2-x}Sr_xMg_2Fe_{12}O_{22}$ refined from room temperature single-crystal X-ray diffraction data. Refinements are performed with B(Fe, O) and O, Fe(1), Fe(4), Fe(5) and Fe(11) occupancies fixed. Fe(12) and Fe(8) sites together contain 2 units of Mg^{2+} to balance charge. Mg/Fe(12) means the Mg quantity on Fe(12) sites. Due to the role of balancing charge and limited volume of the tetrahedral sites, the most probable sites for Mg are in the middle of the big block of Fe(12) and Fe(8) octahedral sites. From the single-crystal X-ray diffraction refinement results, the actual Sr/Ba ratio is slightly lower but consistently increasing as its corresponding nominal Sr level.
